# Local symptoms of Hashimoto’s thyroiditis: A systematic review

**DOI:** 10.3389/fendo.2022.1076793

**Published:** 2023-01-19

**Authors:** Jiaojiao Yuan, Shuo Qi, Xufan Zhang, Hezheng Lai, Xinyi Li, Chen Xiaoheng, Zhe Li, Simiao Yao, Zhiguo Ding

**Affiliations:** ^1^ Dongzhimen Hospital, Beijing University of Chinese Medicine, Beijing, China; ^2^ The First Clinical Medical College, Beijing University of Chinese Medicine, Beijing, China; ^3^ Sunsimiao Hospital, Beijing University of Chinese Medicine, Tongchuan, Shanxi, China; ^4^ National Institute of Complementary Medicine, Western Sydney University, Westmead, NSW, Australia

**Keywords:** Hashimoto’s thyroiditis, symptoms, prevalence, quality of life, hypothyroidism

## Abstract

**Objective:**

Hashimoto’s thyroiditis (HT) is the most common type of thyroid disease and can cause many different manifestations. The local symptoms of HT are an under-studied area of research. Therefore, the purpose of this study was to investigate the local symptoms of HT and their prevalence.

**Methods:**

A systematic review was performed to find articles in PubMed that discuss the local symptoms of HT. Relevant vocabulary terms and key terms included: autoimmune thyroid disease (AITD), hyperthyroidism, hypothyroidism, neck, throat, pharynx, airway, esophagus, breathe, swallow, globus, sleep apnea, symptoms, and quality of life. Two investigators independently screened the eligible studies.

**Results:**

A total of 54 articles fulfilled the inclusion criteria. Of these, 25 were clinical studies, 24 were case reports, and five were reviews. These clinical studies and case reports included a total of 2660 HT patients. There were eight local symptoms related to HT: neck pain (0.02%~16%), voice changes (7%~30%), throat discomfort (20%~43.7%), shortness of breath (28%~50%), dysphagia (29%), goiter-related symptoms (69.44%), sleep apnea, and generally defined compressive symptoms. Due to the use of different outcome measures among all the studies, a meta-analysis of the data could not be performed.

**Conclusion:**

Goiter symptoms, which are an item on the ThyPRO scales, are the most frequent local symptoms in HT patients, and include neck pain, voice changes, throat discomfort, and dysphagia. These local symptoms should be identified in the clinic and included in the early diagnosis and management of HT, as well as evaluated further to understand their relevance in the pathogenesis of HT.

## 1 Introduction

Hashimoto’s thyroiditis (HT) is considered the most common disease among autoimmune thyroid diseases (AITDs), and occurs in approximately 0.3~1.5/1000 subjects/year (1). Pathologically, lymphoplasmacytic infiltration, lymphoid follicle formation with germinal centers, and parenchymal atrophy are typical histopathologic features (2). The pathological diagnosis of HT is based on lymphocytic infiltration on cytological examination (2). Clinically, approximately 25-30% of patients have thyroid dysfunctions (3). Most HT patients with euthyroid or hyperthyroid ultimately evolve into hypothyroidism (3). In most cases, the clinical diagnosis of HT is based on the characteristics, including positivity to serum antibodies against thyroid antigens (thyroid peroxidase and thyroglobulin) (3). The mainstream treatment is focused on the management of hypothyroidism with thyroxine substitution therapy (2).

Generally, the clinical features, which include both local and systemic manifestations of HT, are not typical in patients during the early stage (3). Several articles reported that local manifestations, such as throat problems, dysphagia, neck swelling, and pain, eventually developed when the thyroid gland became enlarged (1). The systemic manifestations were more common in patients with thyroid dysfunction. Patients with hyperthyroidism tended to have palpitations, tremors, heat intolerance, sweating, anxiety, disturbed sleep, weight loss, and polydipsia (4). Patients with hypothyroidism tended to have fatigue, lethargy, cold intolerance, weight gain, constipation, and dry skin (5). There has been a lot of research that focuses on systemic symptoms in patients with thyroid dysfunction. In contrast, the types of local symptoms and their prevalence in HT patients is an under-researched area. This study reviewed the literature to investigate the local symptoms of HT and their prevalence.

## 2 Methods

### 2.1 Data sources and searches

A systematic review was performed in PubMed to search for published studies from 1996 to August 28^th^, 2022. MeSH words, titles, abstracts, and key terms were searched. The search strategy followed was: 1) Hashimoto thyroiditis, autoimmune thyroiditis, autoimmune thyroid disease, hyperthyroidism, thyroid peroxidase antibody (TPOAb), thyroglobulin antibody (TGAb), and thyroid microsomal antibody (TMAb); 2) neck, pain, swollen, throat, pharynx, airway, esophagus, breathe, dyspnea, swallow, globus, dysphagia, and sleep apnea; 3) symptoms, sign, manifestation, quality of life, and ThyPRO; 4) 1 AND (2 OR 3).

### 2.2 Eligibility criteria

Inclusion criteria included clinical trials, case reports, and reviews, with descriptions of specific prevalence or prevalence of local symptoms around HT patients’ neck, including neck, throat, pharynx, airway, esophagus, et al. Studies were excluded that: (a) did not relate to specified search terms in the title or abstract, (b) did not include HT or AITD in the title or abstract, (c) did not illustrate local symptoms of HT in the entire article, (d) were not included in the Science Citation Index (SCI), € publications in languages other than English, and (f) where the full text was unavailable.

### 2.3 Study selection

Study selection was performed independently by two reviewers (JY and XL). In the first phase, all identified titles and abstracts were evaluated for relevance in thyroid disease. In the second phase, potentially relevant articles were subsequently selected and evaluated through full-text screening. The relevance of the articles in reporting the prevalence or specific prevalence of local symptoms in HT was finally included. In case of a disagreement, a third experienced reviewer (SQ) was consulted to achieve a consensus.

### 2.4 Data extraction

The following variables in the included studies were extracted: article type, publication date, author, country, region, IF and Quartile of articles, number of cases of HT, age, gender, thyroid function, and prevalence of cases with local symptoms.

## 3 Results

### 3.1 Search findings

**Figure 1:**
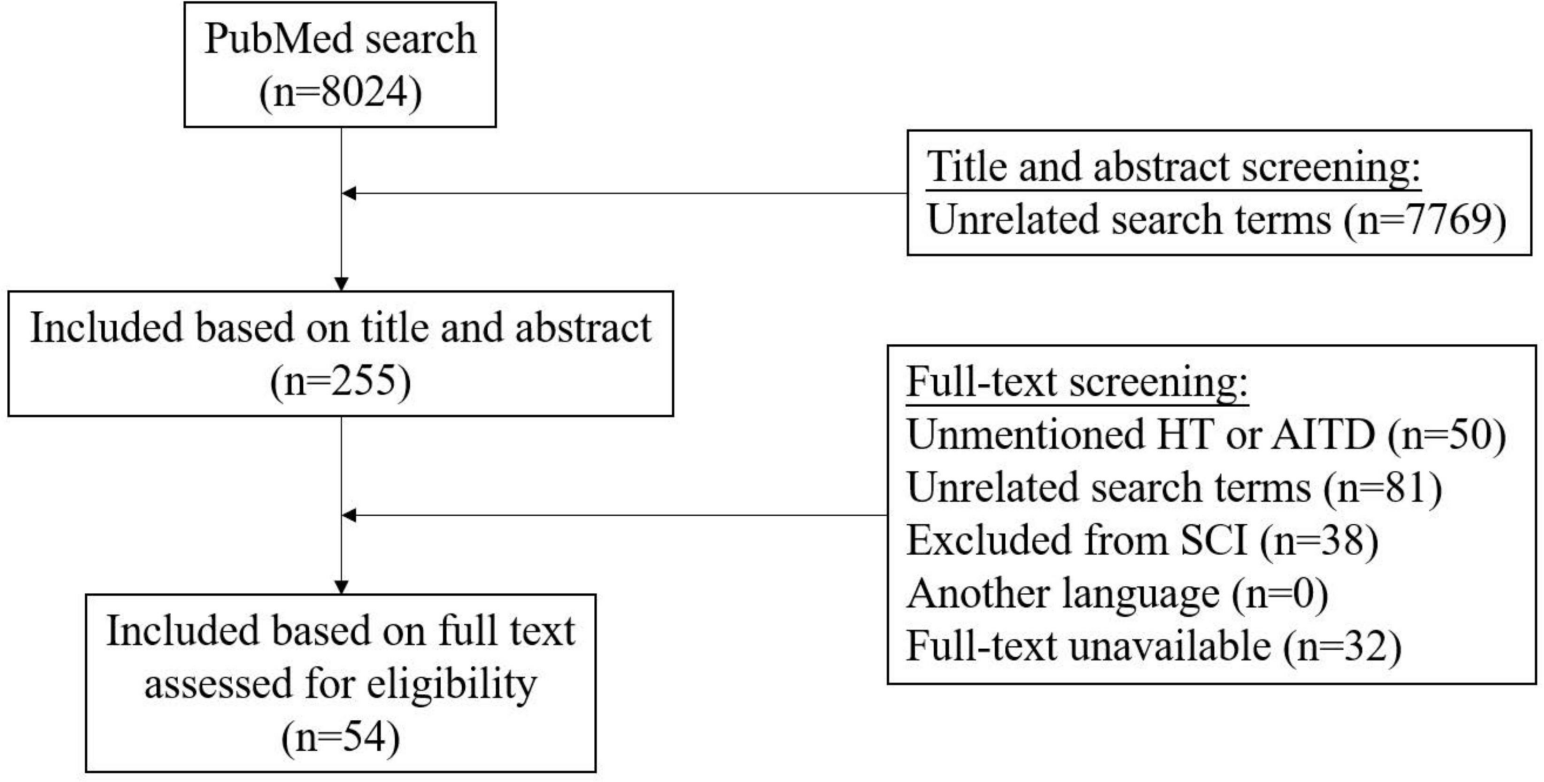
Flowchart illustrating the results of the literature-search performed in this systematic review.

The PubMed search was performed and initially yielded 8024 records. We excluded 7970 articles through title and abstract, and full-text screening (n = 7769, and n = 201, respectively) (**Figure 1**).

### 3.2 Study characteristics

Of the 54 studies included in the review, 25 were clinical studies, 24 were case reports, and five were reviews. After full-text review, it was found that 29 studies mentioned hypothyroidism, five mentioned hyperthyroidism, six mentioned euthyroid, and 15 were unclear about the thyroid function of objects in the articles (**Table 1**).

**Table 1:** Study characteristics.

Thyroid function	Clinical studies	Case reports	Reviews	Total
**Hypothyroidism**	9	18	2	29
**Hyperthyroidism**	1	4	0	5
**Euthyroid**	4	1	1	6
**Unclear**	8	4	3	15
**Total**	**25**	**24**	**5**	**54**

### 3.3 Distribution of local symptoms

**Figure 2:**
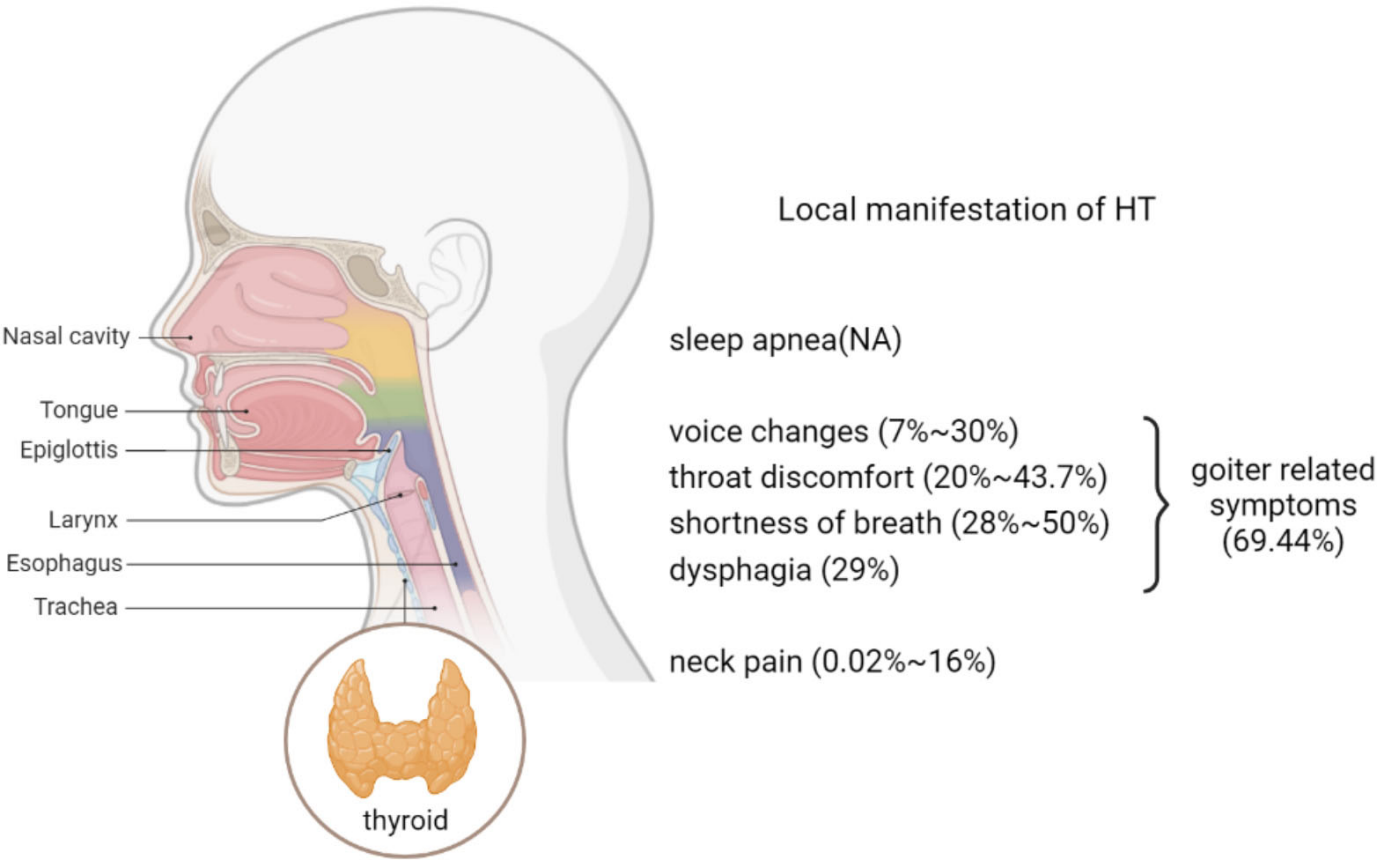
Prevalence of local symptoms.

The local symptoms of HT described in all the selected literature were classified into eight categories: neck pain (16 articles), voice changes (11 articles), throat discomfort (eight articles), dyspnea (eight articles), dysphagia (six articles), goiter-related symptoms (seven articles), sleep apnea (six articles), and unclear compressive symptoms (four articles). There were more than two different local symptoms in several studies (**Table 2**). The prevalence is listed in **Figure 2**.

**Table 2:** Distribution of local symptoms.

Symptoms	Clinical studies	Case reports	Reviews	Total
**Neck pain**	4	10	2	16
**Voice changes**	8	3	0	11
**Throat discomfort**	7	1	0	8
**Dyspnea**	3	5	0	8
**Dysphagia**	2	4	0	6
**Sleep apnea**	3	2	1	6
**Goiter symptoms**	6	0	2	8
**Compressive symptoms**	4	0	0	4
**Total**	**25**	**24**	**5**	**54**

#### 3.3.1 Neck pain

There were 10 case reports and four clinical studies included, with a total of 645 HT patients, comprising 456 cases of hypothyroidism, 34 cases of hyperthyroidism, and 52 cases of euthyroid. The prevalence of neck pain in HT patients, especially in hypothyroidism in the clinical studies, was between 0.02% ~ 16% (**Table 3**).

**Table 3:** Neck pain.

No.	Article Publication year	Region	IF	Quartile	Patients	Age (year)	Sex (male/female)	Thyroid function (number of patients)	Prevalence (%)	Article type
1	Ichijo2020 (6)	Japan	1.271	Q4	1	30	0/1	Hyperthyroidism (1)	–	Case reports
2	Krakovitz2018 (7)	USA	3.325	Q1	5	14.8	1/4	Hypothyroidism (3), euthyroid (2)	–	Case reports
3	Tamborinipermunian2017 (8)	Italy	3.339	Q3	1	47	0/1	Hypothyroidism (1)	–	Case reports
4	Visser2012 (9)	Netherlands	6.568	Q1	1	69	0/1	Hypothyroidism (1)	–	Case reports
5	Ohye2006 (10)	Japan	1.271	Q1	4	48.25	0/4	Hyperthyroidism (1)	–	Case reports
6	Kon2003 (11)	USA	5.958	Q2	7	34.29	0/7	Hypothyroidism (4),unclear (3)	–	Case reports
7	Konno2002 (12)	Japan	4.256	Q2	1	71	0/1	Hypothyroidism (1)	–	Case reports
8	Gourgiotis2002 (13)	USA	3.443	Q4	2	44	1/1	Hypothyroidism (2)	–	Case reports
9	Shigemasa1990 (14)	Japan	5.958	Q2	8	47 ± 9	1/7	Hypothyroidism (1), hyperthyroidism(2), unclear (5)	–	Case reports
10	Leung1989 (15)	Canada	3.183	Q2	1	13	0/1	Hypothyroidism (1)	–	Case reports
11	Carlé2021 (16)	Denmark	4.965	Q2	140	None	None	Hypothyroidism (140)	16%	Clinical study
12	Krakovitz2018 (7)	USA	3.325	Q1	305	None	None	Hypothyroidism (305)	0.02%	Clinical study
13	Thomas2014 (17)	India	2.375	Q4	144	34.18 ± 12.71	15/129	Hypothyroidism (64), euthyroid (50), hyperthyroidism (30)	4.86%	Clinical study
14	Shih2008* (18)	China Taiwan	6.568	Q1	25	42.0(34-81)	0/25	Hypothyroidism (22), unclear (3)	33.3%*	Clinical study
15	Rotondi2017 (19)	Italy	4.256	Q2	–	–	–	Unclear	–	Review
16	Kasagi2006 (20)	Japan	1.271	Q4	–	–	–	Unclear	–	Review

*: the patients in the study were prepared to take a thyroidectomy.

None: the specific detail in the study was unclear.

#### 3.3.2 Voice changes

There were three case reports and eight clinical studies included, with a total of 1005 HT patients, comprising 378 cases of hypothyroidism, zero cases of hyperthyroidism, and 582 cases of euthyroid. The prevalence of voice changes in HT patients across six different regions was between 7% ~ 30% (**Table 4**).

**Table 4:** Voice changes.

No.	Article Publication year	Region	IF	Quartile	Patients	Age (year)	Sex (male/female)	Thyroid function (number of patients)	Prevalence (%)	Article type
1	Kotwal2016 (21)	India	2.309	Q4	1	40	1/0	Hypothyroidism (1)	–	Case reports
2	Monica2012 (22)	Italy	3.25	Q3	1	84	0/1	Hypothyroidism (1)	–	Case reports
3	Hosako-Naito1999 (23)	Japan	1.538	Q4	1	48	0/1	Hypothyroidism (1)	–	Case reports
4	Carlé2021 (16)	Denmark	4.965	Q2	140	None	None	Hypothyroidism (140)	30%	Clinical study
5	Barić2019 (24)	Croatia	3.657	3	290	37.89	20/270	Euthyroid (290)	7%	Clinical study
6	Lima2017 (25)	Brazil	6.292	Q2	117	43.16	23/190	Hypothyroidism (213)	25.35%	Clinical study
7	Rhee2015 (26)	Korea	2.153	3	56	40.3	6/50	Unclear (56)	Unclear	Clinical study
8	Banks2012 (27)	USA	3.325	Q1	29	None	None	Unclear (29)	Unclear	Clinical study
9	McManus2012* (28)	USA	2.192	Q4	311	46 ± 1	31/280	Euthyroid (292), unclear (19)	13%*	Clinical study
10	Heman-Ackah2011 (29)	USA	2.009	Q3	34	None	None	Unclear (34)	Unclear	Clinical study
11	Shih2008* (18)	China Taiwan	6.568	Q1	25	42.0(34-81)	0/25	hypothyroidism22、unclear3	25%*	Clinical study

*: the patients in the study were prepared to take a thyroidectomy.

None/unclear: the specific detail in the study was unclear.

#### 3.3.3 Throat discomfort

Included were one case report and seven clinical studies, with a total of 393 HT patients, comprising 158 cases of hypothyroidism, zero cases of hyperthyroidism, 49 cases of euthyroid, and 152 cases of unclear thyroid function. The prevalence of throat discomfort in HT patients was between 20%~35% (**Table 5**).

**Table 5:** Throat discomfort.

No.	Article Publication year	Region	IF	Quartile	Patients	Age (year)	Sex (male/female)	Thyroid function (number of patients)	Prevalence (%)	Article type
1	Kamienski2007 (30)	USA	2.22	Q4	1	45	0/1	Hypothyroidism (1)	–	Case reports
2	Fukuhara2021 (31)	Japan	1.641	Q4	4	None	None	Unclear (4)	Unclear	Clinical study
3	Carlé2021 (16)	Denmark	4.965	Q2	140	None	None	Hypothyroidism (140)	35%	Clinical study
4	Karahatay2015 (32)	Turkey	3.507	Q4	92	51.16 ± 16.08	25/67	Unclear (92)	20%	Clinical study
5	Banks2012* (27)	USA	3.325	Q1	29	None	None	Unclear (29)	Unclear	Clinical study
6	Greenblatt2009* (33)	USA	3.352	Q3	31	None	None	Hypothyroidism (17)	Unclear	Clinical study
7	Bazzichi2007 (34)	Italy	2.98	Q3	49	52.76 ± 12.42	1/48	Euthyroid (49)	43.7%	Clinical study
8	Grabe2005 (35)	Germany	6.392	Q1	47	49.3 ± 13.2	2/45	Unclear (27)	Unclear	Clinical study

*: the patients in the study were prepared to take a thyroidectomy.

None/unclear: the specific detail in the study was unclear.

#### 3.3.4 Dyspnea

There were five case reports and three clinical studies included, with a total of 172 HT patients, comprising 168 cases of hypothyroidism, one case of hyperthyroidism, zero cases of euthyroid, and three cases of unclear thyroid function. Two clinical studies from two different regions showed that the occurrence of dyspnea in HT patients was 12.5% ~ 50%. One study presented the prevalence of shortness of breath in HT patients with hypothyroidism at 50% (**Table 6**).

**Table 6:** Dyspnea.

No.	Article Publication year	Region	IF	Quartile	Patients	Age (year)	Sex (male/female)	Thyroid function (number of patients)	Prevalence (%)	Article type
1	Gong2017 (36)	China	2.57	Q4	1	28	1/0	Hyperthyroidism (1)	–	Case reports
2	Mohamed2014 (37)	Australia	39.89	Q1	1	67	0/1	Hypothyroidism (1)	–	Case reports
3	Vargas2014 (38)	Italy	5.562	Q1	1	75	1/0	Hypothyroidism (1)	–	Case reports
4	Reynolds2006 (39)	UK	2.299	Q4	2	newborn	0/2	Hypothyroidism (2)	–	Case reports
5	Laurent2004 (40)	France	5.958	Q2	1	35	0/1	Hypothyroidism (1)	–	Case reports
6	Carlé2021 (16)	Denmark	4.965	Q2	140	None	None	Hypothyroidism (140)	short of breath 50%、wheezing28%	Clinical study
7	Liang2020 (41)	China	3.932	Q4	None	None	None	Unclear	None	Clinical study
8	Shih2008* (18)	China Taiwan	6.568	Q1	25	42.0(34-81)	0/25	Hypothyroidism (22), unclear (3)	12.5%*	Clinical study

*: the patients in the study were prepared to take a thyroidectomy.

None: the specific detail in the study was unclear.

#### 3.3.5 Dysphagia

There were four case reports and two clinical studies included, with a total of 169 HT patients, comprising 164 cases of hypothyroidism, one case of hyperthyroidism, and four cases of unclear thyroid function. The prevalence of dysphagia in HT patients with hypothyroidism was 29%. The prevalence of dysphagia in the patients who were prepared to take a thyroidectomy was 33.3% (**Table 7**).

**Table 7:** Dysphagia.

No.	Article Publication year	Region	IF	Quartile	Patients	Age (year)	Sex (male/female)	Thyroid function (number of patients)	Prevalence (%)	Article type
1	Laurent2004 (40)	France	5.958	Q2	1	35	0/1	Hypothyroidism (1)	–	Case reports
2	Guldiken2006 (42)	Turkey	1.264	Q4	1	70	0/1	Hyperthyroidism (1)	–	Case reports
3	Pereira2000 (43)	Brazil	6.568	Q1	1	20	1/0	Hypothyroidism (1)	–	Case reports
4	Shawker1981 (44)	USA	3.959	Q2	1	63	0/1	Unclear (1)	–	Case reports
5	Carlé2021 (16)	Denmark	4.965	Q2	140	None	None	Hypothyroidism (140)	29%	Clinical study
6	Shih2008* (18)	China Taiwan	6.568	Q1	25	42.0(34-81)	0/25	Hypothyroidism (22), unclear (3)	33.3%*	Clinical study

*: the patients in the study were prepared to take a thyroidectomy.

None/unclear: the specific detail in the study was unclear.

#### 3.3.6 Sleep apnea

There were two case reports and four clinical studies included, with a total of 144 HT patients, comprising 16 cases of hypothyroidism, two cases of hyperthyroidism, 106 cases of euthyroid, and four cases of unclear thyroid function. The prevalence of dysphagia in HT patients with hypothyroidism was 29%. However, none of the studies recorded the prevalence of sleep apnea in HT patients (**Table 8**).

**Table 8:** Sleep apnea.

No.	Article Publication year	Region	IF	Quartile	Patients	Age (year)	Sex (male/female)	Thyroid function (number of patients)	Prevalence (%)	Article type
1	Eloy2007 (45)	USA	6.568	Q1	1	59	0/1	Unclear (1)	–	Case reports
2	Stöllberger2001 (46)	Austria	1.808	Q3	1	43	1/0	Hypothyroidism (1)	–	Case reports
3	Sriphrapradang2019 (47)	Thailand	2.816	Q3	33	None	None	Euthyroid (16), hypothyroidism (15), hyperthyroidism (2)	unclear	Clinical study
4	Bozkurt2012 (48)	Turkey	2.394	Q4	106	None	None	Euthyroid (106)	unclear	Clinical study
5	Sakellaropoulou2011 (47)	Greece	3.219	Q4	3	9.5	1/2	unclear3	unclear	Clinical study
6	Xerfan2019 (49)	Brazil	4.062	Q3	–	–	–	unclear	unclear	Clinical study

None/unclear: the specific detail in the study was unclear.

#### 3.3.7 Goiter symptoms (ThyPRO scales)

There were eight articles included, and six clinical studies, with a total of 761 HT patients, comprising 736 cases of hypothyroidism, 32 cases of hyperthyroidism, and 74 cases of euthyroid. One article showed the prevalence of goiter symptoms in HT patients was 69.44% (**Table 9**).

**Table 9:** Goiter symptoms.

No.	Article Publication year	Region	IF	Quartile	Patients	Age (year)	Sex (male/female)	Thyroid function (number of patients)	Prevalence (%)	Article type
1	Morón-Díaz2021 (50)	Spain	3.633	Q3	124	54(45-62)	31/187	Hypothyroidism (124)	unclear	Clinical study
2	Winther2016 (51)	Denmark	3.24	Q3	78	47(18-91)	8/70	Hypothyroidism (78)	unclear	Clinical study
3	Zivaljevic2015 (52)	Serbia	6.701	Q3	27	None	None	Euthyroid (24), hyperthyroidism (1), hypothyroidism (2)	unclear	Clinical study
4	Thomas2014 (17)	India	2.375	Q4	144	34.18 ± 12.71	15/129	Hypothyroidism (64), euthyroid (50), hyperthyroidism (30)	69.44%	Clinical study
5	Watt2014 (53)	Denmark	4.147	Q2	189	None	None	Hypothyroidism (189)	unclear	Clinical study
6	Watt2012 (54)	Denmark	4.209	Q4	199	44(19-88)	15/184	Hypothyroidism (199)	unclear	Clinical study
7	Groenewegen2021 (55)	Netherlands	7.094	Q2	–	–	–	hypothyroidism,euthyroid	–	Review
8	Feller2018 (56)	Switzerland	56.272	Q1	–	–	–	hypothyroidism	–	Review

None/unclear: the specific detail in the study was unclear.

#### 3.3.8 Compressive symptoms

Four clinical studies showed compressive symptoms, but none of these described specific manifestations. A total of 761 HT patients were in these clinical studies, with 736 cases of hypothyroidism, 32 cases of hyperthyroidism, and 74 cases of euthyroid. These studies were limited to HT patients who were to undergo thyroid surgery, and the symptom prevalence was 34%* ~ 72.4%* (**Table 10**).

**Table 10:** Compressive symptoms.

No.	Article Publication year	Region	IF	Quartile	Patients	Age (year)	Sex (male/female)	Thyroid function (number of patients)	Prevalence (%)	Article type
1	Banks2012* (27)	USA	3.325	Q1	29	None	None	Unclear (29)	72.4%*	Clinical study
2	McManus2011* (28)	USA	2.192	Q4	311	46 ± 1	31/280	Euthyroid (292), Unclear (19)	63%*	Clinical study
3	Wormer2011* (57)	USA	2.565	Q3	216	None	None	Unclear (216)	34%*	Clinical study
4	Shih2008* (18)	China Taiwan	6.568	Q1	25	42.0(34-81)	0/25	Hypothyroidism (22), Unclear (3)	62.5%*	Clinical study

*: the patients in the study were prepared to take a thyroidectomy.

None/unclear: the specific detail in the study was unclear.

## 4 Discussion

HT is a common thyroid disease with clinical features that include both local and systemic manifestations (3). These manifestations occur not only under the conditions of hypothyroidism or hyperthyroidism but also under euthyroidism (55).

Thyroid-related symptoms are the specific symptoms related to thyroid disease. In past studies, thyroid-related symptoms were initially described as systemic symptoms, such as cold/heat intolerance, dry skin, unexplained hair loss, chronic constipation, recent weight change, fatigue, depression, mood swings, increased stress, and restlessness (58). However, local thyroid-related symptoms were under-studied, with a dearth of systematic review literature that explores this area, especially in HT.

In our review, we specifically examined the local symptoms in HT patients. The percentage of HT patients who experienced goiter symptoms (17), which included symptoms of the neck, throat, breathing, and swallowing, was 69.44%. Goiter symptoms, which mostly resulted in goiter thyroid glands, are important parts of the Thyroid-specific Patient Reported Outcome (ThyPRO) questionnaire. The ThyPRO questionnaire was developed and implemented as the international standard measurement of thyroid-related QoL of patients with benign thyroid diseases. Its reliability, validity, and responsiveness have been extensively documented in many countries (53). In the ThyPRO questionnaire, goiter symptoms are described as the following 11 symptoms: the sensation of fullness in the neck, a visible swelling in front of the neck, pressure in the throat, pain in the throat, pain in the neck, the sensation of a lump in the throat, frequent throat clearing, discomfort swallowing, difficulty swallowing, a sensation of suffocation, and hoarseness. These annoying symptoms can affect the quality of life and mental health of patients with HT to varying degrees (53).

It was reported that 20% ~ 43.7% of HT patients experienced symptoms of throat discomfort (32, 34). Most HT patients with throat symptoms initially attended an ENT clinic. Morinaka (53, 54) found that patients with thyroid disease accounted for about 1.8% of ENT outpatients, and approximately 53.5% of these thyroid disease patients had HT. Therefore, a thyroid examination could be considered necessary for ENT patients and other patients that exhibit symptoms of throat discomfort. Globus is a sensation of having a lump or foreign body in the throat, accounting for approximately 4% of new otorhinolaryngology outpatients (59). In addition to thyroid nodules and enlarged thyroid volume, the inflammation of the thyroid may be another factor reducing uncomfortable throat symptoms (32).

Symptoms of dyspnea were experienced in 28% ~ 50% of HT patients, including chest tightness, wheezing (28%), shortness of breath (50%), and suffocation (16). The difference in the prevalence of the respective specified symptoms is related to the severity of airway obstruction.

Nearly 7% ~ 30% of HT patients experienced voice changes, which included voice deepening, hoarseness, and dysphonia (16–18). Approximately 29% of HT patients experienced dysphagia (16). Immuno-mediated diseases may affect the laryngeal function and vocal fold vibration through multi-factorial mechanisms, while dysphonia or dysphagia may be the first sign of an autoimmune disease (60). Galli (60) investigated the prevalence and severity of dysphonia, globus pharynges, and dysphagia in patients affected by immune-mediated (IM) diseases by the Voice Handicap Index (VHI) (scale 0-4) and Glasgow-Edinburgh Throat Scale (GETS). The self-assessment questionnaires could be considered a useful tool for early detection of vocal dysfunction in HT patients, to prevent further deterioration of quality of life and serious life-threatening complications (61).

It was discovered that 0.02% ~ 16% of HT patients experienced pain in the front of their neck (7, 16, 17). Painful Hashimoto thyroiditis (pHT) is an atopic subtype of HT characterized by acute, progressive, unbearable pain on one side or the entire thyroid area (19). Some hypotheses suggested that pHT is caused by capsular stretching with rapid enlargement of the thyroid (13). However, pain could also emerge in atrophic thyroid glands and other thyroid-disease patients, such as in DeQuervain thyroiditis, acute suppurative thyroiditis, hemorrhage in the thyroid nodule, and in the rapid growth of the thyroid tumor (19), making it necessary for a differential diagnosis.

Most local manifestations originate from compression of the cervical structures that are anatomically close to the thyroid gland, and include dysphonia (from the involvement of the recurrent laryngeal nerve), dyspnea (from the restriction of the trachea), and dysphagia (from impingement upon the esophagus) (3). Therefore, several studies considered that HT patients with aggressive compressive symptoms, such as dyspnea, dysphagia, unrelievable pain, and globus, could experience relief after a thyroidectomy (11, 18). A few studies suspected that the immune disorder may be a risk factor for local manifestations (55, 60).

Several studies found that there is a certain correlation between levels of thyroid-related antibodies and the occurrence of local symptoms. Watt et al. (53) found a positive correlation between TPOAb and goiter symptoms (p=0.019, r=0.17). Other studies suggested that TPOAb levels are associated with an increased prevalence of airflow limitation or asthma (62). However, there is a lack of studies that indicate whether a relief of goiter symptoms occurs after effective treatment to decrease TPOAb.

In addition to the above symptoms, there are also some changes in local signs, such as goiter (enlarged neck and disappearance of necklines), and enlarged cervical lymph nodes. Some studies found that the prevalence of cervical lymphadenopathy in patients with positive TPOAb or TGAb is approximately 63% ~ 88.5% (63–65), and they suggested that cervical lymphadenopathy may be one of the indicators to distinguish thyroiditis from other benign thyroid diseases.

From the 54 studies in our review, local manifestations were usually ignored in the clinic and research. However, they could affect the quality of life and mental health of HT patients. According to the literature, a thyroidectomy is an option to relieve the aggressive compressed symptoms. However, the necessity of surgical treatment remains to be discussed. Thus, diversified treatment methods to improve local manifestations could play an important role in the management of HT and in delaying the process of hypothyroidism.

## 5 Limitations

There were some limitations identified in this review. First, this review included clinical research and case reports that discussed the differences in local symptoms between HT patients and patients suffering from other benign thyroid diseases. Thus, these findings may not reflect the real-world prevalence of these local symptoms in HT patients. Second, most symptoms in the research were subjective and lacked certain definitions. Third, this review also included some cross-sectional studies that focused on one or two of these local symptoms and the results of which cannot be representative of the comprehensive local symptoms of HT. Lastly, this review included some studies of HT patients who were prepared to undertake a thyroidectomy or had hypothyroidism, which may create bias in the prevalence of the local symptoms. Therefore, our findings of the prevalence of local symptoms through this review are considered as an initial basis for reference, and a more extensive and in-depth cross-sectional investigation is needed to clarify the epidemiological characteristics of local symptoms of HT.

## 6 Conclusions

HT patients do experience local symptoms, such as pain, voice changes, throat discomfort, dyspnea, dysphagia, and sleep apnea. The goiter symptoms in the ThyPRO scales include most of these local symptoms, and thus the ThyPRO is a good questionnaire to evaluate the symptoms of HT. At present, HT patients with aggressive compressive symptoms have been recommended for a thyroidectomy in some studies. The necessity of surgical treatment remains to be discussed. The presentation of local symptoms of HT should be identified in clinics for improvement in the detection and early diagnosis and management of HT, as well as evaluated further to understand their relevance in the pathogenesis of HT.

## Author contributions

All authors listed have made a substantial, direct, and intellectual contribution to the work and approved it for publication.
